# Analytical platform evaluation for quantification of ERG in prostate cancer using protein and mRNA detection methods

**DOI:** 10.1186/s12967-015-0418-z

**Published:** 2015-02-12

**Authors:** Jintang He, Athena A Schepmoes, Tujin Shi, Chaochao Wu, Thomas L Fillmore, Yuqian Gao, Richard D Smith, Wei-Jun Qian, Karin D Rodland, Tao Liu, David G Camp, Anshu Rastogi, Shyh-Han Tan, Wusheng Yan, Ahmed A Mohamed, Wei Huang, Sreedatta Banerjee, Jacob Kagan, Sudhir Srivastava, David G McLeod, Shiv Srivastava, Gyorgy Petrovics, Albert Dobi, Alagarsamy Srinivasan

**Affiliations:** Biological Sciences Division, Pacific Northwest National Laboratory, Richland, WA USA; Environmental Molecular Sciences Laboratory, Pacific Northwest National Laboratory, Richland, WA USA; Center for Prostate Disease Research, Department of Surgery, Uniformed Services University of the Health Sciences, Bethesda, MD USA; Cancer Biomarkers Research Group, Division of Cancer Prevention, National Cancer Institute, Bethesda, MD USA; Urology Service, Walter Reed National Military Medical Center, Bethesda, MD USA

**Keywords:** ERG, Quantification, Biomarker, PRISM-SRM, MRM, Mass spectrometry, ELISA, Prostate cancer, Diagnosis

## Abstract

**Background:**

The established methods for detecting prostate cancer (CaP) are based on tests using PSA (blood), PCA3 (urine), and AMACR (tissue) as biomarkers in patient samples. The demonstration of ERG oncoprotein overexpression due to gene fusion in CaP has thus provided ERG as an additional biomarker. Based on this, we hypothesized that ERG protein quantification methods can be of use in the diagnosis of prostate cancer.

**Methods:**

An antibody-free assay for ERG3 protein detection was developed based on PRISM (high-pressure high-resolution separations with intelligent selection and multiplexing)-SRM (selected reaction monitoring) mass spectrometry. We utilized *TMPRSS2-ERG* positive VCaP and *TMPRSS2-ERG* negative LNCaP cells to simulate three different sample types (cells, tissue, and post-DRE urine sediment). Enzyme-linked immunosorbent assay (ELISA), western blot, NanoString, and qRT-PCR were also used in the analysis of these samples.

**Results:**

Recombinant ERG3 protein spiked into LNCaP cell lysates could be detected at levels as low as 20 pg by PRISM-SRM analysis. The sensitivity of the PRISM-SRM assay was approximately 10,000 VCaP cells in a mixed cell population model of VCaP and LNCaP cells. Interestingly, ERG protein could be detected in as few as 600 VCaP cells spiked into female urine. The sensitivity of the in-house ELISA was similar to the PRISM-SRM assay, with detection of 30 pg of purified recombinant ERG3 protein and 10,000 VCaP cells. On the other hand, qRT-PCR exhibited a higher sensitivity, as *TMPRSS2-ERG* transcripts were detected in as few as 100 VCaP cells, in comparison to NanoString methodologies which detected *ERG* from 10,000 cells.

**Conclusions:**

Based on this data, we propose that the detection of both *ERG* transcriptional products with RNA-based assays, as well as protein products of *ERG* using PRISM-SRM assays, may be of clinical value in developing diagnostic and prognostic assays for prostate cancer given their sensitivity, specificity, and reproducibility.

## Background

Human cancers are commonly stratified by expression status and/or mutation of cancer causing genes and their encoded proteins. Genetic rearrangements involving DNA sequences from different chromosomes or intrachromosomal regions have been extensively documented in various cancers, including prostate cancer (CaP) [1]. It has been reported that several *ETS* transcription factors play important roles in CaP as a result of genetic rearrangements. Of these, overexpression of the *ETS*-related gene (*ERG*) [2,3], resulting from the fusion of *ERG* coding sequences to the androgen-responsive *TMPRSS2* gene [4], represents the most common subtype, with a prevalence of approximately 50% in clinically localized prostate cancers [1,5-11]. In addition, studies evaluating the expression of *ERG* in matched benign and malignant prostate tissues from a large patient cohort indicated that CaP cells harboring *TMPRSS2-ERG* fusions showed overexpression of *ERG* in 60-70% of patients [8]. This genomic rearrangement is now established as one of the most common mechanisms of oncogenic activation in CaP [6,9,12]. *ERG* overexpression has also been implicated in a diverse number of cancers, including Ewing’s sarcoma and acute myeloid leukemia [13-15].

A major goal in CaP is to define protein and antibody markers which may facilitate early detection, distinguish indolent from aggressive disease, define treatment strategies, and allow follow up of patients. The prevalence of *ERG* overexpression has therefore provided an impetus for the development of detection assays for *TMPRSS2-ERG* mRNA in cells from tissues or urine samples from CaP patients [16,17]. Currently, real-time quantitative reverse transcription PCR (qRT-PCR), which detects the presence of *TMPRSS2-ERG* fusion transcripts, is routinely used in research and clinical laboratories. However, the selection of primer-probe sets used for evaluation has resulted in variable sensitivity in the detection of the respective RNA. This has led to the development of monoclonal and polyclonal antibodies for the detection of ERG protein for diagnostic and/or therapeutic purposes [18-20]. In this regard, a mouse monoclonal antibody (MAb) against ERG was developed in our laboratory. One of the ERG MAb clones, 9FY, recognized an epitope formed by the amino acid sequence GQTSKMSPRVPQQDWLSQPPARVTI, which corresponds to residue positions 42-66 in the ERG protein [NCBI Reference Sequence: NP_891548.1] [18,21]. The 9FY monoclonal antibody was found to be highly specific in the detection of ERG protein in cell culture-based experiments and human prostate cancer specimens by immunofluorescence and immunohistochemistry (IHC), respectively, without cross-reactivity to other members of the *ETS* family [18,20]. Similar observations were also reported for a rabbit monoclonal antibody using the C-terminal peptide of ERG as an immunogen [19,22].

Recent analysis of whole mount prostate sections from age and pathologic stage matched specimens from over 180 patients revealed that there is a striking difference in ERG expression in African American and Caucasian American patients [20]. Much lower frequencies (10-27%) of *ERG* alterations have been reported in studies from China, Japan, and India [23-26]. This overexpression of ERG protein in prostate cancer cells may result in a scenario in which the protein may also be released in body fluids, either through a non-classical secretory pathway and/or lysis of cells, providing ERG as a marker associated with the distinct stage of the disease.

While IHC is ideal for the analysis of biopsied tissues from patients, assays to quantitate ERG protein are desirable for the analysis of cells in blood and urine samples. As there are no commercially available serologic assays for ERG, there is a need to develop assays that are sensitive, accurate, and offer the flexibility of testing multiple target proteins simultaneously. Emerging targeted proteomic technologies, exemplified by the selected reaction monitoring mass spectrometry (SRM-MS), are ideal for achieving these goals with high multiplexing capability and good reproducibility [27-29]. However, a major limitation of SRM-based targeted quantification is the lack of sufficient sensitivity for measuring low abundance proteins. To address this issue, we recently developed an antibody-independent strategy, termed high-pressure high-resolution separations with intelligent selection and multiplexing (PRISM), for significantly enhancing the SRM sensitivity by at least 100-fold when compared to conventional liquid chromatography (LC)-SRM [30-32]. More recently, PRISM-SRM assays have been utilized for accurately measuring 16 distinct peptides from various domains of *TMPRSS2-ERG* gene fusion products in prostate cancer cell lines and patient-derived tumor tissues [33].

In the present study we have systematically evaluated the performance of multiple platforms for the sensitive quantification of ERG expression at the protein and mRNA levels: PRISM-SRM, enzyme-linked immunosorbent assay (ELISA), and western blot assays for ERG protein; qRT-PCR and NanoString assays for *TMPRSS2-ERG* mRNA. In our knowledge, this is the first time a comparative, unbiased evaluation of different platforms analyzing a similar source of samples has been performed for the assessment of ERG. We utilized *TMPRSS2-ERG* positive VCaP and *TMPRSS2-ERG* negative LNCaP cells to simulate three different sample types (cells, tissue, and post-DRE urine sediment) for these protein and mRNA measurements. Our results demonstrate that different analytical platforms vary in their ability to quantify ERG protein or mRNA, and provide a basis for *ERG* testing in patient samples for diagnostic and prognostic purposes.

## Materials and methods

### Cell culture

Vertebral-Cancer of the Prostate (VCaP) cells, Lymph Node Carcinoma of the Prostate (LNCaP), and human embryonic kidney 293 (HEK293) cells were obtained from American Type Culture Collection (ATCC, Manassas, VA). VCaP and HEK293 cells were cultured in Dulbecco’s Modified Eagle Medium (DMEM; ATCC) supplemented with 10% fetal bovine serum (FBS; ATCC). LNCaP cells were cultured in Roswell Park Memorial Institute-1640 medium (RPMI-1640; Life Technologies, Carlsbad, CA) supplemented with 10% FBS. All cells were cultured in humidified conditions, at 37°C with 5% CO_2_.

### Sample preparation

Recombinant full length ERG protein, produced in mammalian cells by exogenous expression, was purchased from Origene (Rockville, MD). HEK293, LNCaP, and VCaP cells were harvested by trypsinization, diluted in PBS, and counted by using a hemocytometer. HEK293 and LNCaP cells were aliquoted at 1,000,000 cells/ml into Eppendorf tubes and pelleted with or without the addition of VCaP cells. Three sample types were prepared: 1) Purified recombinant ERG was incrementally spiked into 1,000,000 LNCaP or HEK293 cell lysates which are negative for *TMPRSS2-ERG*; 2) *TMPRSS2-ERG* fusion positive VCaP cells were titrated into 1,000,000 LNCaP cells incrementally, and pelleted by centrifugation; 3) VCaP cells were incrementally spiked into 5 mL urine donated by female volunteers, collected as per an approved IRB protocol. All samples were prepared at CPDR, and used by both PNNL and CPDR for analyses of ERG.

### PRISM-SRM protein extraction and digestion

Proteins were extracted from sample types 1 and 2 (see above) directly, using a urea solution (8 M urea in 50 mM NH_4_HCO_3_); sample type 3 was first centrifuged at 4,000 × *g* for 45 min in an Amicon Ultra-4 concentrator with 10 kDa molecular weight cutoff (EMD Millipore, Billerica, MA), followed by protein extraction from the concentrated sample using the same urea solution. Cells were sonicated for 1 min, chilled on ice for 1 min, which was repeated for a total of three times, and the protein concentration was determined using the bicinchoninic acid (BCA) assay (Pierce, Rockford, IL). Proteins in each sample were reduced with 10 mM dithiothreitol at 37°C for 1 h and alkylated using 40 mM iodoacetamide at room temperature for 1 h in the dark. Samples were then diluted 10-fold with 50 mM NH_4_HCO_3_ and 1 M CaCl_2_ was added to each sample to reach a final concentration of 1 mM. Protein digestion was performed at 37°C for 3 h using trypsin (Affymetrix, Santa Clara, CA) at a 1:50 enzyme-to-substrate ratio (w/w). Each sample was desalted using a Discovery DSC-18 C18 SPE column (SUPELCO, Bellefonte, PA) and concentrated to a volume of ~50 μL. The peptide concentration was measured using the BCA assay.

### SRM assay development

Seven proteotypic peptides of ERG protein were selected and stable isotope-labeled heavy peptides with C-terminal [^13^C_6_^15^N_2_] lysine or [^13^C_6_^15^N_4_] arginine were synthesized (ThermoFisher Scientific, Waltham, MA) for SRM assay development [33]. SRM parameters were optimized by direct infusion experiments on a TSQ Quantum Ultra triple quadrupole mass spectrometer (ThermoFisher Scientific), where the 6-8 most intense fragment ions for each peptide were selected as precursor-to-fragment transitions and the collision energy (CE) of each transition was optimized automatically in SRM mode. The peptides were dissolved in a buffer containing 50% acetonitrile and 0.1% formic acid, and the infusion rate was 300 nL/min. The transitions and corresponding optimal CE values from the infusion experiments were further validated for optimal detection of the target peptides in actual LC-SRM analysis. In this step 50 fmol/μL of heavy peptide standards were spiked with 0.5 μg/μL of VCaP-derived tryptic peptides, and 2 μL of the sample was analyzed using a nanoACQUITY UPLC® system (Waters Corporation, Milford, MA) and a TSQ Vantage triple quadrupole mass spectrometer (ThermoFisher Scientific). Transitions with lower intensity or higher level of interference were removed, and the three best transitions were retained for each peptide in developing the final SRM assays.

### PRISM fractionation

Five fmol/μL of high-purity heavy peptides (purity > 97%) were spiked with 1 μg/μL of peptides from each sample, and the peptides were separated following the PRISM workflow using high pH reversed-phase capillary LC on a nanoACQUITY UPLC® system as described previously [30,33]. Briefly, separations were performed using a capillary column packed in-house (3 μm Jupiter C18 bonded particles, 200 μm i.d. × 50 cm long) at a flow rate of 3.3 μL/min on binary pump systems, using 10 mM ammonium formate (pH 10) as mobile phase A and 10 mM ammonium formate in 90% acetonitrile (pH 10) as mobile phase B. Forty-five microliters of each sample (1 μg/μL) were loaded onto the column and separated using a binary gradient of 5-15% B in 15 min, 15-25% B in 25 min, 25-45% B in 25 min, and 45-90% B in 38 min. Following the LC separation, the eluate from the capillary column was split into two flowing streams (1:10 split) via a T-union. The smaller fraction of eluate was sent at a flow rate of 300 nL/min to a TSQ Quantum Ultra triple quadrupole mass spectrometer for on-line SRM monitoring of heavy peptide standards. TSQ Quantum Ultra was operated with ion spray voltages of 2400 ± 100 V, a capillary offset voltage of 35 V, a skimmer offset voltage of −5 V, and a capillary inlet temperature of 220°C. Tube lens voltages were obtained from automatic tuning and calibration without further optimization. Both Q1 and Q3 were set at unit resolution of 0.7 FWHM and Q2 gas pressure was 1.5 mTorr. A scan width of 0.002 *m/z* and a dwell time of 10 ms were used. The remaining fraction of the capillary column eluate, flowing at a rate of 3 μL/min, was automatically collected every 1 min into a 96-well plate using a Triversa NanoMate® system (Advion BioSciences, Ithaca, NY) over the course of ~100 min LC separation. Prior to peptide fraction collection, 17 μL of water was added to each well in the plate to avoid peptide loss and also to dilute the peptide fraction for LC-SRM analysis. The fraction containing a target peptide was intelligently selected based on the retention time of the peptide obtained by on-line monitoring. The detailed method for intelligent selection was described in our previous study [30,32].

### LC-SRM analysis

Following high pH capillary reversed-phase LC (RPLC) separation and intelligent selection, the fractions containing the target peptides were subjected to conventional LC-SRM analysis. All peptide fractions were analyzed using a nanoACQUITY UPLC® system coupled on-line to a TSQ Vantage triple quadrupole mass spectrometer. The UPLC® system was equipped with a nanoACQUITY UPLC BEH 1.7 μm C18 column (100 μm i.d. × 10 cm), which was connected to a chemically etched 20 μm i.d. fused-silica emitter via a stainless steel union. Four microliters of each peptide fraction were loaded onto the column at a flow rate of 1 μL/min for 5 min. Peptides were separated at a flow rate of 500 nL/min, using a 10-min gradient from 10-35% acetonitrile in water. The TSQ Vantage was operated in the same manner as the TSQ Quantum Ultra. The raw data acquired on the TSQ Vantage triple quadrupole MS were imported into Skyline software [34] for visualization of chromatograms of target peptides and to quantify the detected peptides. The most abundant transition for each peptide was used for quantification unless interference was observed. Peak detection and integration were based on two criteria: 1) the same retention time; and 2) approximately the same relative peak intensity ratios across multiple transitions between light peptide and heavy peptide standards. All data were manually inspected to ensure correct peak detection and accurate integration. Light to heavy peak area ratios were used to quantify target peptides.

### ELISA assay

An ELISA was developed for use in detection of ERG protein in biospecimens, including body fluids such as sera and urine. Purified recombinant ERG protein was used as a source of antigen for development. Different combinations of antibodies were used as capture and detection reagents in an antigen capture assay, or sandwich ELISA, to select for optimal reactivity. ELISA procedures using cell lysates were carried out in NUNC 96-well flat bottom Maxisorp plates (Thermo Scientific, Rockford, IL). Plates were coated with 1 μg/mL of ERG MAb 9FY, using 100 μL coating buffer (50 mM NaHCO_3_, pH 9.6). The reactions were carried out in duplicate. The plates were covered with microplate sealers (Thermo Scientific) and incubated at 4°C overnight. The next day, plates were washed 4 times with wash buffer (1X PBS + Tween-20; KD Medical, Columbia, MD) and blocked with 200 μL blocking buffer (StartingBlock; Thermo Scientific), covered, and incubated for 1 h at room temperature (RT). Each set of cell samples were lysed through 5 freeze-thaw cycles in a dry ice/methanol bath, followed by sonication at 35% amplitude with 15 s pulses for 10 min. Following pre-clearing by centrifugation (13,000 × *g* for 10 min), lysates (100 μL) were loaded onto plates after washing once post-blocking, covered, and incubated for 1 h at 37°C. Plates were washed 4 times with wash buffer, and incubated with 100 μL biotinylated ERG antibody (2 μg/mL; Origene), covered for 1 h at 37°C. Plates were again washed 4 times with wash buffer and then incubated with 100 μL of Streptavidin-HRP conjugated antibody diluted as per manufacturer’s protocols (KPL Inc., Gaithersburg, MD), covered for 1 h at 37°C. Plates were washed 4 times with wash buffer, 100 μL of K-Blue Aqueous TMB substrate (Neogen, Lexington, KY) was added to the plates, and incubated uncovered for 30 min at RT. Sulfuric acid (2 N, 100 μL) was added to the plates post-incubation to stop the reactions. Plates were immediately read at 450 nm to measure absorbance. All dilutions of reagents were performed in ELISA diluent (20% NGS in 1X PBS with 0.1% Triton-X 100).

### Western blot

VCaP, LNCaP, and HEK293 cells were trypsinized and washed twice with PBS. VCaP cells were counted on a Coulter cell counter and aliquoted in appropriate quantities. Cells were pelleted by centrifugation and pellets were lysed in Mammalian Protein Extraction Reagent (M-PER; Thermo Scientific). Following pre-clearing by centrifugation (13,000 × *g* for 10 min), protein concentrations of cell lysates were determined by using Protein Assay Reagent (Bio-Rad, Hercules, CA). LNCaP and HEK293 cell lysates were spiked with purified recombinant ERG protein at the indicated concentrations, and then both sets of lysates were separated on NuPAGE Bis-Tris (4-12%) gels (Life Technologies) and transferred onto PVDF membranes. Membranes were blocked in Blocking Buffer (LI-COR, Lincoln, NE) and incubated with specific antibodies against ERG (ERG MAb 9FY; Biocare Medical Inc., Concord, CA), and GAPDH (Santa Cruz biotechnology, Santa Cruz, CA). Membranes were washed in Tris-Buffered Saline + Tween 20 (TBST) before incubation with appropriate secondary antibodies (goat anti-Mouse IRDye 800CW or goat anti-Rabbit IRDye 680CW, LI-COR). Signals of proteins detected were visualized and quantitatively measured using the Odyssey infra-red imaging scanner and software (LI-COR).

### Quantitative RT-PCR

RNA was isolated from cell pellets using RNeasy RNA Isolation Kit (Qiagen, Germantown, MD). The total amount of RNA isolated was used for reverse transcription (RT) for a final volume of 20 μL of cDNA, and real-time qRT-PCR (TaqMan) was performed using 5 μL of the cDNA. TaqMan primers and probe for quantitative evaluation of *ERG3* were as follows: forward: 5′-CAGGTCCTTCTTGCCTCCC-3′; reverse: 5′-TATGGAGGCTCCAATTGAAACC-3′; probe: 5′-FAM-TGTCTTTTATTTCTAGCCCCTTTTGGAACAGGA-TAMRA-3′. The expression of glyceraldehyde 3-phosphate dehydrogenase (*GAPDH*) was used as an endogenous control gene in each reaction (Applied Biosystems, Grand Island, NY). RNA samples without reverse transcription were included as negative controls in each assay. Results are represented by the average Ct values from individual runs.

### NanoString platform RNA isolation and assessment

Pellets from VCaP-spiked female urine and VCaP-spiked LNCaP cell mixture were resuspended in 100 μL of extraction buffer. RNA was isolated using a PicoPure RNA isolation kit (Life Technologies), according to manufacturer’s protocols. RNA quality was measured using a Bioanalyzer 2100 (Agilent Technologies, Palo Alto, CA) and a NanoDrop ND-100 spectrophotometer (Thermo Scientific).

### NanoString nCounter analysis

RNA isolated from each sample was input for NanoString nCounter analysis (NanoString Technologies, Seattle, WA). Hybridization and NanoString nCounter were processed according to manufacturer’s protocols reported previously [35,36]. In brief, hybridizations were carried at 65°C for 20 h, after which the hybridization products were applied to the nCounter Preparation Station for automated removal of excess probe and immobilization of probe-transcript complexes on a streptavidin-coated cartridge. Data were collected using the nCounter Digital Analyzer by counting the individual barcodes. Data were analyzed using the nCounter™ digital analyzer software (Version 2.1.1). The raw data were normalized through the following two steps: 1) The geometric mean of spiked-in exogenous positive controls was used to correct for differences resulting from assay efficiency (hybridization, purification, binding, etc.); 2) Spiked-in negative controls were used to remove hybridization background. All signals below mean background +2 standard deviations (SD) were considered as hybridization background and subtracted from the raw data.

## Results

### PRISM-SRM detection of recombinant ERG protein spiked into LNCaP cells

**Figure 1:**
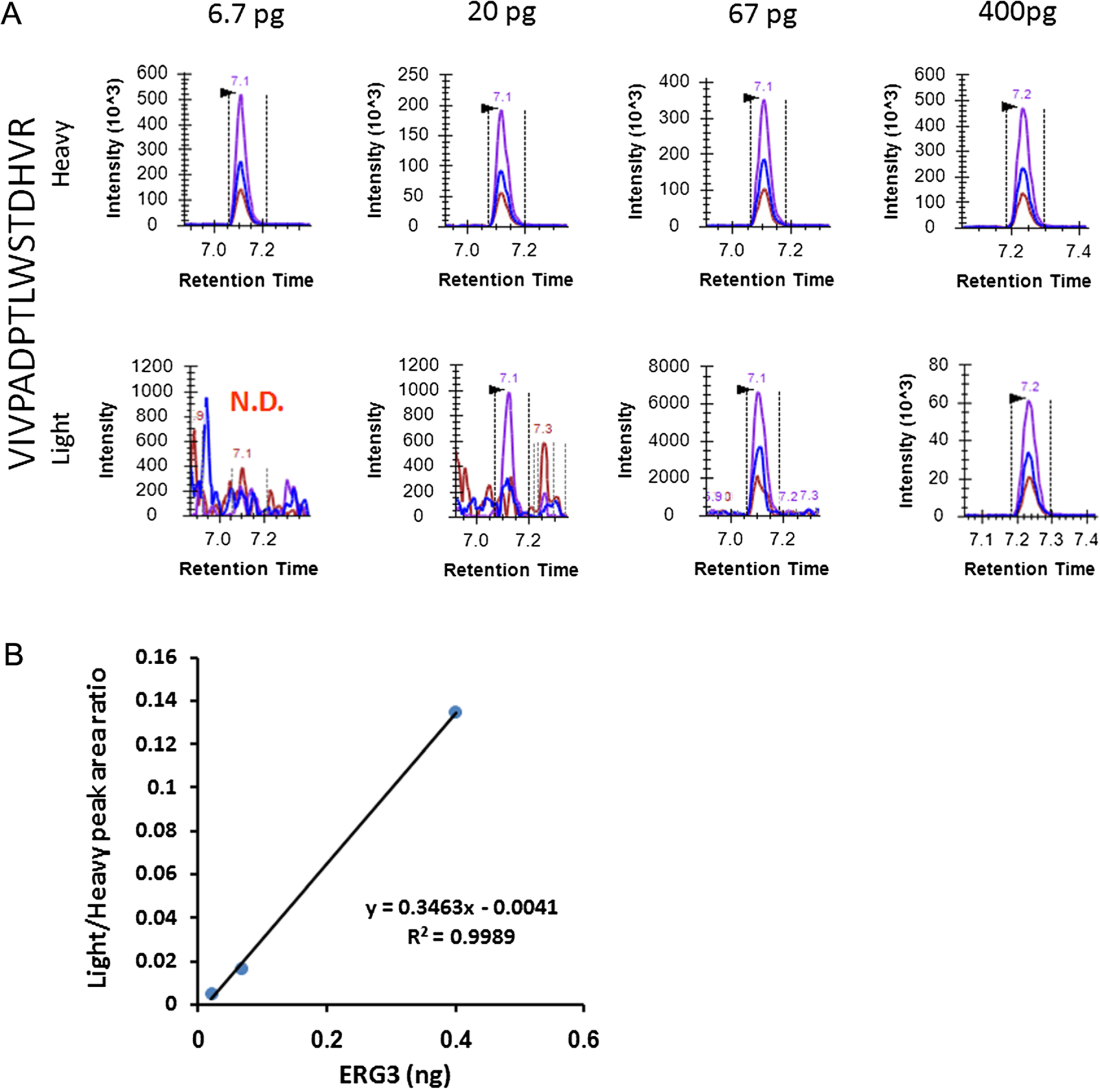
**Detection of ERG peptide VIVPADPTLWSTDHVR from LNCaP cells.** Different amounts of recombinant ERG3 protein (6.7 pg, 20 pg, 67 pg, 400 pg) was spiked into LNCaP cells. **A**. XICs of peptide VIVPADPTLWSTDHVR from the different data points. Heavy indicate heavy internal standard peptide; Light indicate endogenous peptide. N.D.: not detected. **B**. Response curve.

To define ERG detection sensitivity in cells, purified recombinant ERG3 protein (30, 100, 300, 1000, 6000 pg) was spiked into 1,000,000 *TMPRSS2-ERG* negative LNCaP cells and analyzed by PRISM-SRM. After cell lysis, tryptic digestion and sample clean-up, 1/15 of each sample was used for PRISM-SRM analysis. Three ERG peptides were consistently detected, with peptide VIVPADPTLWSTDHVR (residues 125-160 in the predicted amino acid sequence of ERG3) displaying the best response (Table 1). Using this peptide, ERG3 protein could be detected at levels as low as 20 pg (Table 1 and Figure 1A). The response curve showed excellent linearity in the detected spiking range: 300-6,000 pg ERG3 protein spiked into 1,000,000 LNCaP cells (Figure 1B).

**Table 1:** **Detection of recombinant ERG3 protein spiked into LNCaP cells using PRISM-SRM**

**ERG peptide**	**Data point (on-column protein amount)**			
	**6.7 pg**	**20 pg**	**67 pg**	**400 pg**
VIVPADPTLWSTDHVR	ND	√	√	√
HMPPPNMTTNER	ND	ND	√	√
ITTRPDLPYEPPR	ND	ND	√	√

√ indicates that the peptide was detected.

ND indicates that the peptide was not detected.

### PRISM-SRM detection of ERG protein in the VCaP-LNCaP mixed cells

**Figure 2:**
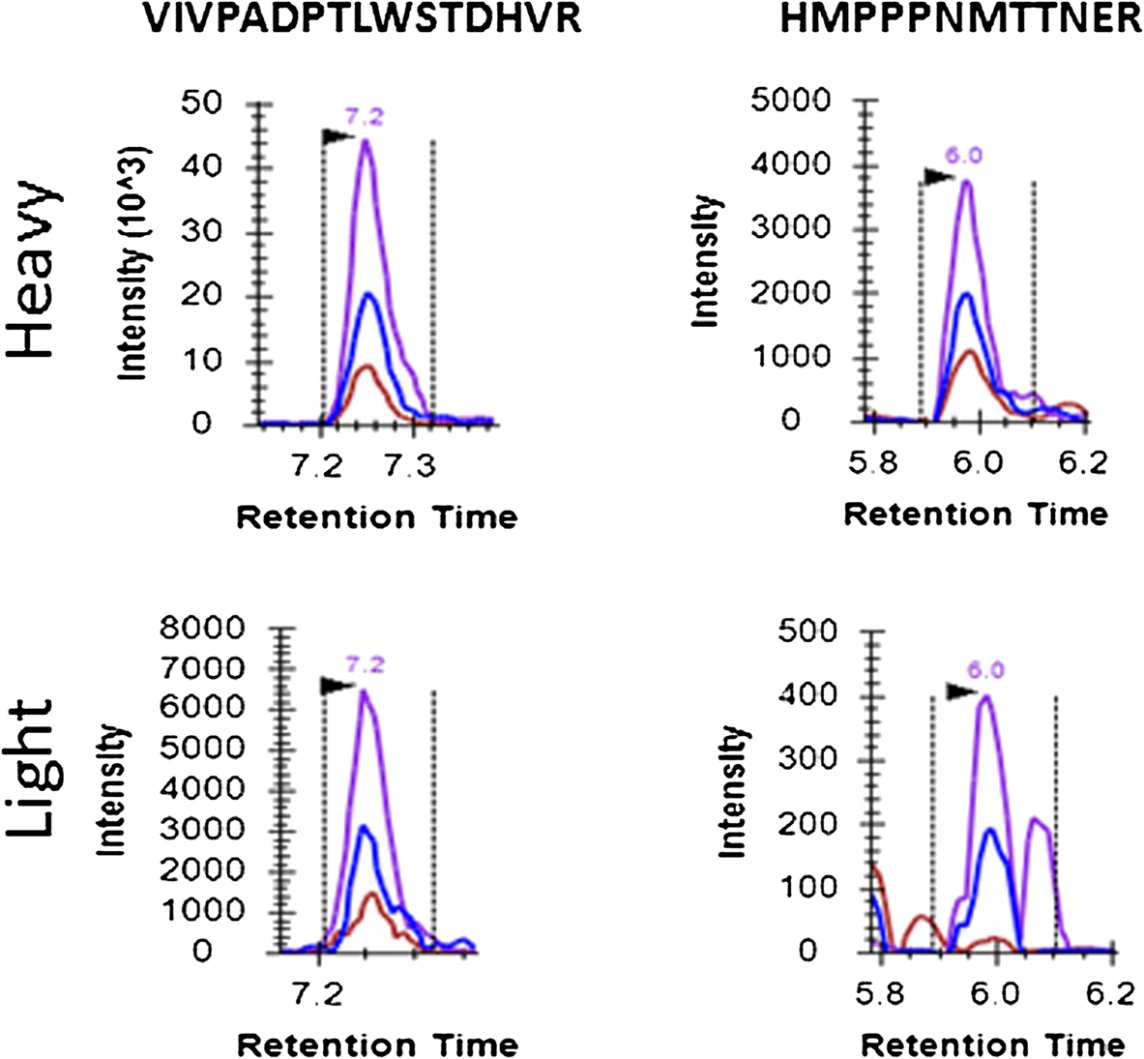
**Detection of ERG protein in the VCaP-LNCaP system.** XICs of the two ERG peptides detected from ~ 10,000 VCaP cells. Heavy indicates heavy internal standard peptide; Light indicates endogenous peptide.

*TMPRSS2-ERG* positive VCaP cells (0, 10, 100, 1,000, 10,000, and 100,000) were incrementally spiked into 1,000,000 LNCaP cells to simulate prostate tumor tissue samples, which contain mixtures of tumor and non-malignant stroma, and 1/10 of each sample was analyzed using PRISM-SRM. In these samples two ERG peptides, VIVPADPTLWSTDHVR (amino acid positions 125-140) and HMPPPNMTTNER (amino acid positions 112-123), were confidently detected from samples corresponding to 10,000 VCaP cells (Table 2). Extracted ion chromatograms (XICs) of the two peptides are shown in Figure 2.

**Table 2:** **Detection of ERG protein in VCaP cells spiked into LNCaP cells using PRISM-SRM**

**ERG peptide**	**Data point (number of VCaP cells)**				
	**1**	**10**	**100**	**1000**	**10000**
VIVPADPTLWSTDHVR	ND	ND	ND	ND	√
HMPPPNMTTNER	ND	ND	ND	ND	√

√ indicates that the peptide was detected.

ND indicates that the peptide was not detected.

### PRISM-SRM detection of ERG protein in the VCaP-urine system

**Figure 3:**
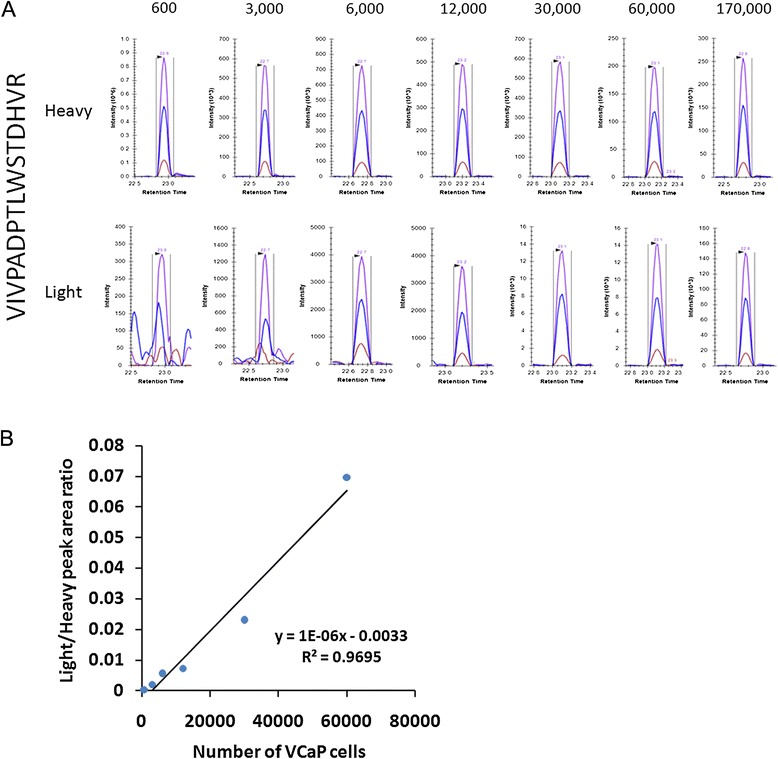
**Detection of ERG peptide VIVPADPTLWSTDHVR in the VCaP-urine system. A**. The number of VCaP cells for each PRISM-SRM analysis data point was 600, 3,000, 6,000, 12,000, 30,000, 60,000, 170,000, respectively. Heavy indicates heavy internal standard peptide; Light indicates endogenous peptide. **B**. The light/heavy peak area ratio shows good linearity.

VCaP cells (0, 1,000, 5,000, 10,000, 20,000, 50,000, 100,000, and 1,000,000) were spiked into 5 mL of human female urine to simulate prostate cancer urine sediment samples, and analyzed using PRISM-SRM. Due to the limited total protein content (i.e., the low yield of urinary proteins from urine samples) in these samples, 60% of each sample (as opposed to 1/10) was used for the analysis, except for the highest concentration data point sample (where 1/6 of the sample was used for the analysis). The results showed that four ERG peptides were detected and peptide VIVPADPTLWSTDHVR had the best response, which is consistent with the above observations (Table 3). XICs of VIVPADPTLWSTDHVR showed that this peptide could be detected and quantified from as low as 600 VCaP cells (Figure 3A). Based on the peak area ratio of this ERG peptide and the concentration of heavy peptide standard, the abundance of ERG protein was estimated as ~18,000 copies per VCaP cell, i.e., 1.8 fg per cell. The response curve of the best detected peptide, VIVPADPTLWSTDHVR, again showed good linearity (Figure 3B).

**Table 3:** **Detection of ERG protein in VCaP cells spiked into human female urine**

**ERG peptide**	**Data point (number of VCaP cells)**							
	**0**	**600**	**3000**	**6000**	**12000**	**30000**	**60000**	**170000**
VIVPADPTLWSTDHVR	ND	√	√	√	√	√	√	√
HMPPPNMTTNER	ND	ND	ND	√	√	√	√	√
MVGSPDTVGMNYGSYMEEK	ND	ND	ND	ND	ND	ND	√	√
ITTRPDLPYEPPR	ND	ND	ND	ND	ND	ND	ND	√

√ indicates that the peptide was detected.

ND indicates that the peptide was not detected.

### Detection of ERG protein by ELISA

**Figure 4:**
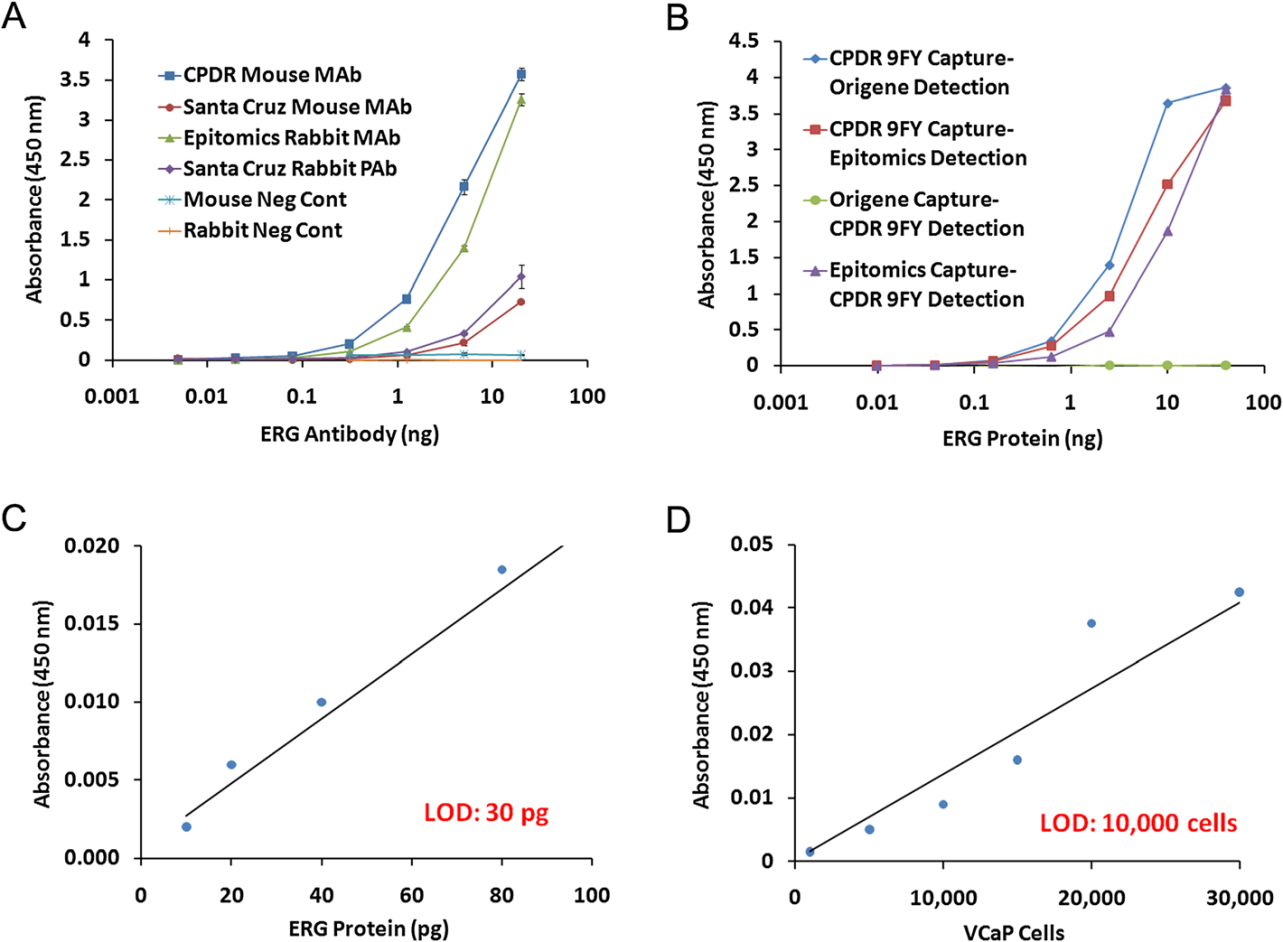
**Detection of ERG protein using ELISA. A**. Evaluation of ERG antibody reactivity in the ELISA format. **B**. Capture and detection antibody combinations for development of antigen capture assay (sandwich ELISA). **C**. The limit of detection of purified recombinant ERG protein was 30 pg. **D**. The limit of detection of ERG protein from VCaP cell lysates (without mixing with LNCaP cells) was 10,000 cells.

For the development of an in-house ELISA for ERG protein detection, we initially tested the affinity of several antibodies available against ERG protein. The reactivity data indicated that the ERG MAb 9FY antibody showed high affinity when compared to antibodies from Epitomics, Origene, and Santa Cruz (Figure 4A). As the sandwich ELISA format requires two antibodies that recognize the ERG protein at non-overlapping sites, we used recombinant ERG protein to test a series of antibody combinations for the capture and detection of ERG. Our results showed that a combination of ERG MAb 9FY for capture and Origene ERG biotinylated antibody (Clone 5 F12) has high sensitivity for detecting ERG protein (Figure 4B). We utilized this specific antibody combination for generating a standard curve for the detection of ERG protein (Figure 4C), as well as defining the limit of detection for ERG, which was found to be 30 pg. The results showed a linear relationship with respect to the concentration of ERG protein and absorbance values noted. A similar linear relationship was observed with recombinant ERG spiked into cell lysates. However, cell lysates from the VCaP-LNCaP mixed cell population did not yield quantifiable ERG data using ELISA methods, presumably due to an overabundance of cellular protein (data not shown). Therefore, we utilized the sandwich ELISA to evaluate detection limit of ERG protein in VCaP cells directly (without mixing with LNCaP cells). The number of VCaP cells considered for analysis included: 1,000, 5,000, 10,000, 15,000, 20,000, 30,000, 40,000, 50,000, 75,000 and 100,000. The results indicated that the limit of detection was 10,000 cells (Figure 4D). Based on the ERG protein quantification results derived from the 20,000-cell data point, the absolute concentration of ERG approximates 9 fg per cell.

### Detection of ERG protein by western blot

**Figure 5:**
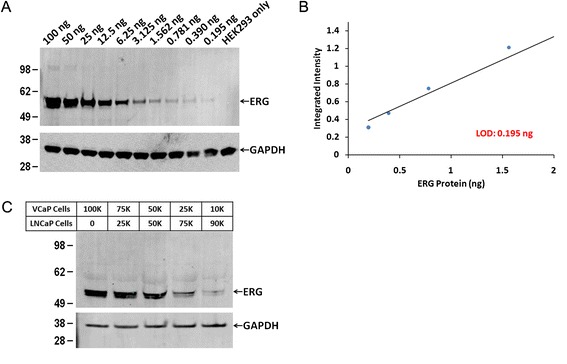
**Western blot analysis for detection of ERG protein in cell lysates. A**. A 55 kDA band corresponding to ERG protein was observed in HEK293 cell lysates spiked with purified recombinant protein in all lanes except the HEK293 only sample. GAPDH was used as a loading control. **B**. The limit of detection was 0.195 ng of ERG protein in western blot analysis. **C**. Cell mixtures containing VCaP and LNCaP cells, kept constant with 100,000 total cells, revealed that 10,000 VCaP cells are detected by western blot. GAPDH was again used as a loading control.

To assess the detection limit of ERG protein by western blot, we used the HEK293 cell line, which lacks expression of *TMPRSS2-ERG*. Recombinant ERG protein was added to 15 μg equivalent of HEK293 cell lysate in the following amounts: 100, 50, 25, 12.5, 6.25, 3.125, 1.562, 0.781, 0.390 and 0.195 ng. As expected, a band corresponding to 55 kDa was noted in all the cell lysates spiked with recombinant ERG protein except for the control (Figure 5A). The intensity of the signal was quantified using Odyssey Infrared Imaging System (LI-COR). The four highest dilutions were presented as scatter plot in Figure 5B. This showed that the limit of detection was 195 pg of the recombinant ERG protein. We then quantified endogenous ERG protein present in VCaP cells (10,000 to 100,000) mixed with ERG negative LNCaP cells (the overall cell number was kept constant at 100,000). The results showed that ERG protein signal was detected in as little as 10,000 VCaP cells (Figure 5C). The analysis of the signals through scanning densitometry revealed that the signal from 10,000 VCaP cells corresponds approximately to 440 pg of ERG based on the standard curve.

### Detection of *ERG3* mRNA by qRT-PCR

**Figure 6:**
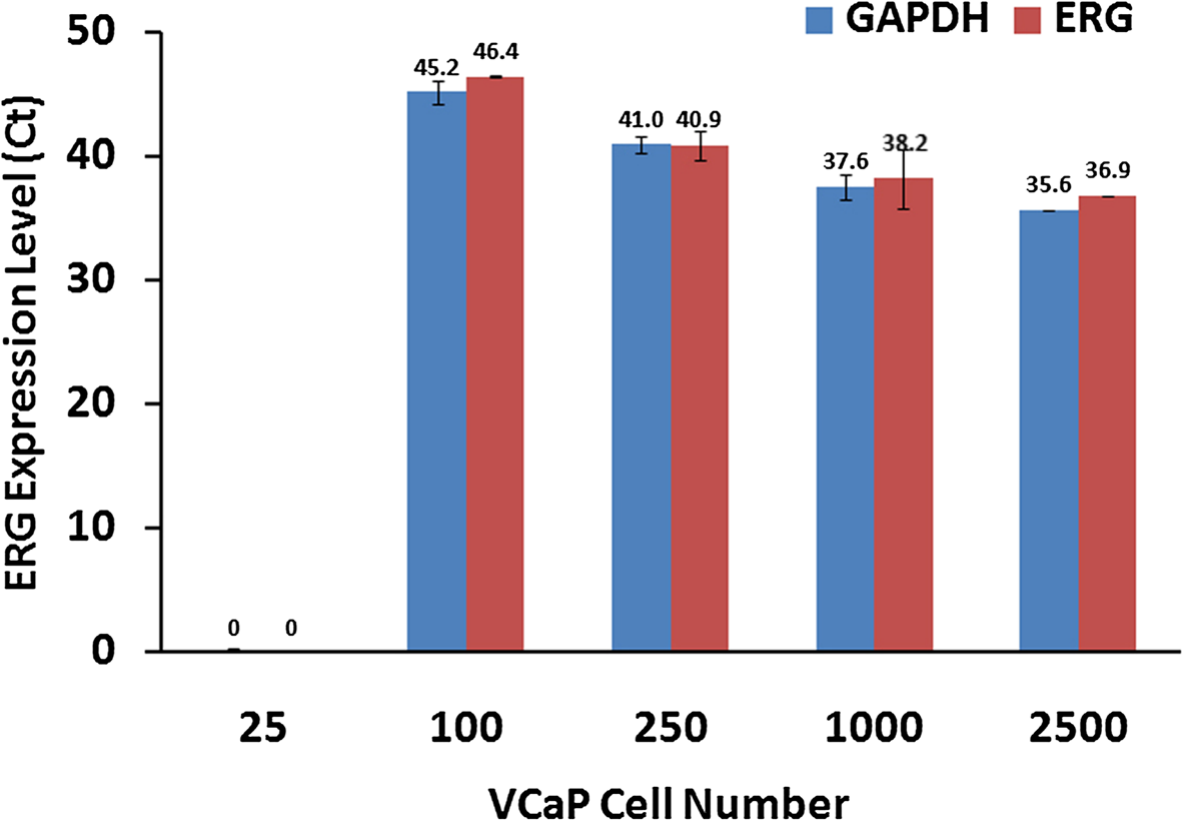
**Real time quantitative RT-PCR evaluation of** ***ERG*** **in VCaP cells spiked into female urine.** mRNA transcripts of *ERG3* were detectable in as few as 100 cells.

In an effort to evaluate the detection limit of *ERG* at the mRNA level with respect to cell number, we utilized *TMPRSS2-ERG* fusion harboring VCaP cells. The VCaP-LNCaP mixed cell population was not amenable for the assay due to high RNA concentrations in a relatively small sample size, thus we utilized urine samples incrementally spiked with VCaP cells. *ERG3,* the longest functional splice form of *ERG*, was consistently detected from cDNA corresponding to as few as 100 VCaP cells. The Ct values of the internal control, *GAPDH*, corresponded with *ERG* values, and both became progressively lower as cell numbers increased (Figure 6).

### Detection of *ERG3* by NanoString

**Figure 7:**
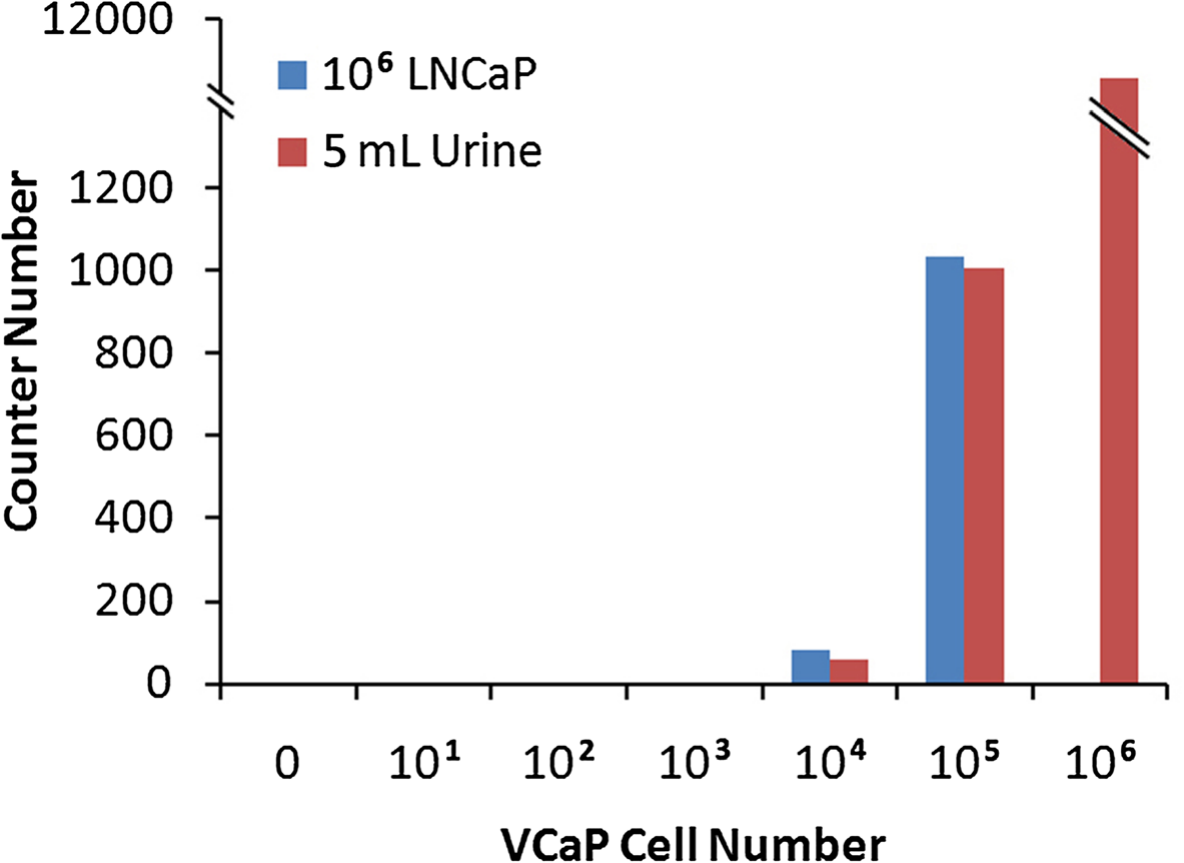
**Detection of** ***ERG*** **using NanoString.** *ERG* copy number was detected from 10,000 VCaP cells spiked into LNCaP cells, as well as VCaP cells spiked into 5 mL of female urine.

RNA extracted from the LNCaP-VCaP mixed cell sample showed high quality RNA with the median RNA integrity number (RIN) of 8.7 (range 8.3-9.0) and 260/280 of 1.84 (range 1.84-2.03), respectively. However, VCaP cells spiked urine samples failed measurement by both the Bioanalyzer (Nanochip) and Nanodrop, showing an undetectable RIN or 260/280 (Table 4). However, all of the RNA isolated from each sample was used for hybridization, therefore, analysis of *ERG* was not affected by RNA assessment and the NanoString readout directly correlated with the initial number of cells in each sample. Figure 7 shows that the NanoString detected the highest level of *ERG* (copy number) in urine spiked with one million VCaP cells (~11,000 copies, lower panel). Around 1,000 copies of *ERG* were detected in both LNCaP cells and urine samples spiked with 100,000 VCaP cells, while 81 and 57 copies of *ERG* were detected in LNCaP cells and urine spiked with 10,000 VCaP cells, respectively (Table 5). Given that the number of RNA copies detected was decreased by almost the identical fold change of VCaP cell numbers, *ERG* should be detected for both LNCaP cells and urine spiked with 1,000 VCaP cells by the NanoString nCounter system, with around 5 copies each. The lack of *ERG* detection from both of these cell preparations might result from our stringent background subtraction in which we removed all signals below mean background + 2 standard deviations (median of SD = 5). Extrapolating from the above results, we should be able to detect around 25 copies of RNA in 5000 VCaP cells (1/2 fold of the number from 10, 000 VCaP cells), by the NanoString from both spiked LNCaP cells and urine.

**Table 4:** **RNA assessment by Bioanalyzer and Nanodrop**

**Sample**	**Bioanalyzer**			**Nanodrop**		
	**ng/mL**	**RIN**	**Total RNA (ng)**	**ng/mL**	**260/280**	**Total RNA (ng)**
10^6^ LNCaP	100	8.3	2800	110	1.93	3080
10^6^ LNCaP + 10^1^ VCaP	540	8.6	15120	513	1.96	14364
10^6^ LNCaP + 10^2^ VCaP	280	8.9	7840	302	1.91	8456
10^6^ LNCaP + 10^3^ VCaP	350	9	9800	393	1.84	11004
10^6^ LNCaP + 10^4^ VCaP	350	8.6	9800	333	2.03	9324
10^6^ LNCaP + 10^5^ VCaP	250	8.8	7000	277	1.9	7756
5 mL Urine	30	ND	840	11	ND	308
5 mL Urine + 10^2^ VCaP	30	ND	840	6	ND	168
5 mL Urine + 10^3^ VCaP	10	ND	280	8	ND	224
5 mL Urine + 10^4^ VCaP	80	ND	2240	9	ND	252
5 mL Urine + 10^5^ VCaP	10	ND	280	22	ND	616
5 mL Urine + 10^6^ VCaP	130	ND	3640	83	ND	2324

ND indicates that a reading was not detected.

**Table 5:** **Detection of ERG in VCaP cells spiked into LNCaP cells and human female urine using NanoString nCounter**

**Spiked Sample**	**Number of VCaP Cells**						
	**0**	**10** ^**1**^	**10** ^**2**^	**10** ^**3**^	**10** ^**4**^	**10** ^**5**^	**10** ^**6**^
10^6^ LNCaP	ND	ND	ND	ND	81.85	1033.8	ND
5 mL Urine	ND	ND	ND	ND	57.55	1004.12	10945.38

ND indicates that a reading was not detected.

## Discussion

The discovery of fusion transcripts involving *ERG* coding sequences and *TMPRSS2* promoter sequences has elucidated a role for *ERG* in the initiation and progression of prostate cancer in a subset of patients. PSA screening has been used as a diagnostic test for prostate cancer since the 1990s. Although this is a convenient assay using blood samples, the specificity of the assay is only around 20% [15,16]. This has led to frequent overtreatment and its related consequences. This scenario has also spurred significant interest in the search for new and highly specific biomarkers for the detection of prostate cancer. In this regard, tests involving various forms of PSA have also been utilized with moderate success.

The detection of ERG protein expression in biopsied tissues from prostate cancer patients using the highly specific ERG MAb 9FY has been valuable for the stratification of prostate cancer. Several laboratories, including ours, have shown that ERG expression is detected only in prostate tumor tissue and not in matched controls, consistent with studies showing low/undetectable levels of expression of endogenous ERG protein in prostate epithelial cells [18,19]. It has also been demonstrated that PRISM-SRM detects ERG protein only in the *TMPRSS2-ERG* fusion positive tumors, and not in the *TMPRSS2-ERG* fusion negative tumors [33]. Recently, we have completed a study involving 1000 prostate whole mount specimens and the results confirmed our earlier observations ([20]; unpublished data). In addition, there is a striking population difference as Caucasian Americans registered *ERG* expression in about 50% of patients compared to a relatively low level of expression in African Americans (25%) [20,23].

The analysis of biopsied tissues is currently carried out by IHC which is labor intensive and, for the most part, qualitative rather than quantitative in nature. Quantitative approaches, based on either a single or a combination of biomarkers, are therefore desirable for the diagnosis and prognosis of prostate cancer. Considering this, we sought to develop assays for assessing the level of ERG oncoprotein in cells from multiple sample types including tissue and urine sediment. Such assays are likely to be useful in clinical applications such as patient stratification. For example, it is well known that prostate cancer cells are present in urine/post-DRE urine samples of patients, due to manual manipulation of the prostate, which represent a truly non-invasive source for analysis [37]. In comparison to analysis involving tissues containing endothelial cells in addition to prostate tumor cells as a source of analysis, the cells present in the post-DRE urine may be mostly devoid of endothelial cells. In such a scenario, the observed ERG signal could be attributed to its expression from tumor cells. Recently, the FDA approved the PCA3 urine test based on RNA evaluation, which is used in the context of making decisions about repeat biopsies [38]. Along the same line, cells in urine samples could be tested for the presence of ERG oncoprotein.

Several different technologies were evaluated for quantification of ERG protein with the goal of identifying their detection limit. Commercially available purified recombinant ERG protein, established prostate cancer cell lines VCaP (*TMPRSS2-ERG* fusion positive) and LNCaP (*TMPRSS2-ERG* fusion negative), and female urine were used to prepare three types of samples mimicking cell lines, tissues, and urine sediments for the analysis. It is worth noting that the antibody-free, MS-based PRISM-SRM assay enables the detection of 20 pg of recombinant ERG protein spiked into LNCaP cell lysate. In a mixed cell population model containing a constant number (1,000,000) of LNCaP cells with an incremental number of VCaP cells, the assay was able to detect and quantify ERG protein in 10,000 VCaP cells. Interestingly, the same assay detected ERG protein in as few as 600 VCaP cells spiked into female urine samples. This sensitivity difference between the mixed cell line samples and the female urine is most likely because the spiked urine sample has less total protein content than the mixed VCaP/LNCaP cell line sample, hence, when the same amount of peptides from these two different sample types were injected for PRISM-SRM analysis, the effective loading of ERG is increased, and the background (i.e., matrix effects) is reduced in the simulated urine sediment samples.

In addition to developing antibody-free assays for the quantification of ERG protein, we have also compared the sensitivity of both protein and RNA detection methods. The detection limit of the former assay, using ERG MAb 9FY as the capture antibody in ELISA, was in the range of 30 pg of recombinant ERG. It should be noted that the 9FY antibody recognizes an epitope at the amino terminus of the ERG protein and has the potential to detect protein encoded by all *TMPRSS2-ERG* variants. The limit of detection for western blot analysis was approximately 195 pg of recombinant ERG protein. Thus, PRISM-SRM at a 20 pg detection limit was the most sensitive of the three methods. With regard to the detection of cells expressing ERG, both ELISA and western blot methods exhibited the sensitivity of detecting 10,000 VCaP cells, the same as the PRISM-SRM assay. The comparison studies were also expanded to include methods detecting mRNA by qRT-PCR and NanoString. Quantitative RT-PCR showed high sensitivity as it was able to detect RNA from a minimum of 100 VCaP cells spiked in female urine. It should be pointed out that our RNA detection method utilized total RNA without involving a pre-amplification step in the analysis. While the NanoString has the advantage of analyzing the expression of multiple genes simultaneously, it was found to be less sensitive than qRT-PCR assay, as it detected RNA from 10,000 VCaP cells spiked into LNCaP cells. Several factors may contribute to the differences in sensitivity with respect to detection limit in the analysis of the mixed VCaP/LNCaP cells and VCaP spiked urine samples. These include: i) dilution of ERG protein in a mixed cell population model; ii) the lysis of cells involving freeze-thaw and sonication methods; iii) differences in the stability and half-life of the analytes; and iv) limitation of the sensitivity of capturing and detection antibodies in ELISA.

## Conclusions

In summary, the data presented here suggest that qRT-PCR and PRISM-SRM platforms are highly sensitive in detecting *TMPRSS2-ERG* transcripts and proteins, respectively. Compared to other RNA and protein detection technologies, the PRISM-SRM assay has several significant advantages: it is not affected by RNA stability, it does not rely on a specific antibody, and it is ideal for isoform-specific detection with high multiplexing capability (e.g., multiplexed quantitative analysis of ERG3 and ERG8 isoforms; data not shown). Therefore, PRISM-SRM assays can be adapted to detect ERG protein in cells present in clinical specimens (e.g., tissue, post-DRE urine, and circulating tumor cells in blood), and cell-free ERG protein present in the blood sera, providing an opportunity for its use in the clinical setting for detection in prostate cancer patients, and define treatment strategies in accordance.

## Abbreviations

CaP: Prostate cancer
ERG: ETS-related gene
PSA: Prostate specific antigen
qRT-PCR: Quantitative reverse transcription polymerase chain reaction
MAb: Monoclonal antibody
IHC: Immunohistochemistry
SRM-MS: Selected reaction monitoring mass spectrometry
PRISM: High-pressure high-resolution separations with intelligent selection and multiplexing
LC: Liquid chromatography
ELISA: Enzyme-linked immunosorbent assay
VCaP: Vertebral-Cancer of the Prostate
LNCaP: Lymph Node Carcinoma of the Prostate
HEK293: Human embryonic kidney 293
DMEM: Dulbecco’s Modified Eagle Medium
FBS: Fetal bovine serum
BCA: Bicinchoninic acid
CE: Collision energy
M-PER: Mammalian Protein Extraction Reagent
TBST: Tris-Buffered Saline + Tween 20
RT: Reverse transcription
GAPDH: Glyceraldehyde 3-phosphate dehydrogenase
XIC: Extracted ion chromatograms
RIN: RNA integrity number
